# Environmental risk of leptospirosis infections in the Netherlands: Spatial modelling of environmental risk factors of leptospirosis in the Netherlands

**DOI:** 10.1371/journal.pone.0186987

**Published:** 2017-10-24

**Authors:** Ente J. J. Rood, Marga G. A. Goris, Roan Pijnacker, Mirjam I. Bakker, Rudy A. Hartskeerl

**Affiliations:** 1 KIT Royal Tropical Institute, Health dept., Amsterdam, The Netherlands; 2 WHO/FAO/OIE and National Collaborating Centre for Reference and Research on Leptospirosis, Amsterdam, The Netherlands; Leibniz-Institut fur Pflanzengenetik und Kulturpflanzenforschung Gatersleben, GERMANY

## Abstract

Leptospirosis is a globally emerging zoonotic disease, associated with various climatic, biotic and abiotic factors. Mapping and quantifying geographical variations in the occurrence of leptospirosis and the surrounding environment offer innovative methods to study disease transmission and to identify associations between the disease and the environment. This study aims to investigate geographic variations in leptospirosis incidence in the Netherlands and to identify associations with environmental factors driving the emergence of the disease. Individual case data derived over the period 1995–2012 in the Netherlands were geocoded and aggregated by municipality. Environmental covariate data were extracted for each municipality and stored in a spatial database. Spatial clusters were identified using kernel density estimations and quantified using local autocorrelation statistics. Associations between the incidence of leptospirosis and the local environment were determined using Simultaneous Autoregressive Models (SAR) explicitly modelling spatial dependence of the model residuals. Leptospirosis incidence rates were found to be spatially clustered, showing a marked spatial pattern. Fitting a spatial autoregressive model significantly improved model fit and revealed significant association between leptospirosis and the coverage of arable land, built up area, grassland and sabulous clay soils. The incidence of leptospirosis in the Netherlands could effectively be modelled using a combination of soil and land-use variables accounting for spatial dependence of incidence rates per municipality. The resulting spatially explicit risk predictions provide an important source of information which will benefit clinical awareness on potential leptospirosis infections in endemic areas.

## Introduction

Leptospirosis is a globally emerging disease with numerous outbreaks being reported worldwide over the past decades [1,2]. The pathogen is transmitted by exposure to contaminated water or urine from infected animals and may survive for days to months in freshwater, soil, or mud [3–6]. Livestock farming and agricultural practices are generally believed to be important factors driving endemic leptospirosis transmission leading to human infections [6–10]. The pathogen is endemic in rodent reservoir populations across Europe as well as tropical America, Australia, Asia and Africa, making it a pan-global threat to public health [10]. Small mammals, notably rats, are the most important reservoirs, but also pet and farm animals, living in close vicinity of humans present potential high risk reservoirs [11].

The interplay between *Leptospira* occurrence, reservoir host abundance, reservoir infection rates and local environments is believed to affect the emergence of the disease [1,11]. Leptospirosis endemicity has been associated with wet tropical environments where epidemic outbreaks of the disease are strongly driven by seasonal variations or natural disasters [6,12]. In temperate climates, leptospirosis dynamics show seasonal dynamics, with increased numbers of infections during warmer months [5,6,13]. Climate conditions and persistence of high leptospirosis prevalence in reservoir hosts have been suggested to be amongst the foremost factors leading to increased transmission of leptospirosis across different geographical regions and eco-regions [6]. Yet, the causal pathway by which local environments facilitate the transmission of leptospirosis and how these are altered by varying climate regimes remain largely unknown. This apparent gap in our understanding of how local environments can facilitate transmission, poses a disparity to halt transmission.

This study aims to describe and explain geographic variations in endemic leptospirosis morbidity in the Netherlands. Leptospirosis occurs at an average yearly incidence of 0.25 cases/100,000 population and a case fatality rate of 6.5%. Yet, the occurrence of the disease is geographically highly heterogeneous. Spatial variations in the morbidity of leptospirosis are mapped and the contribution of biotic and abiotic environmental factors and endogenous spatial dispersal processes are investigated and quantified. By explicitly considering the spatial variations in the occurrence of leptospirosis infections the scale, intensity and scale of transmission of the disease are quantified.

## Materials and methods

### Data collection

#### Case data

For this study, retrospective epidemiological data of leptospirosis patients in the Netherlands during the period 1995–2012 were used. Data were collected from archived records from the National Leptospirosis Reference Laboratory. Cases confirmed by the microscopic agglutination test (MAT), IgM ELISA and/or, culture and/or PCR were considered in this study. Case reports were entered into a database including case age, place of residence, date of onset of illness and a short description of the likely place of infection. Cases that were infected outside the Netherlands were excluded from the study.

#### Geocoding

Each confirmed case of leptospirosis was geocoded using the latitude and longitude of residential address of a patient, which were derived using Google Earth v7.0 (2013 Google Inc.). If information on the probable location of infection was known this was the preferred location to geocode a case. The resulting spatial points were projected into the Dutch national Cartesian coordinate system (Rijksdriehoekstelsel Amerfoort). To assess spatial variations in risk rates, case data were aggregated by municipality and population corrected incidence rates were calculated. Aggregating data had the added advantage that the effect of geographical inaccuracies in geolocations resulting from a mismatch between residence and location of infection was reduced. For each municipality, period incidence rates over the period 1995–2012 were calculated based on the municipality population size in the year 2000. To reduce the effect of inflation of incidence rates due to small population sizes, raw incidence rates were transformed using an Empirical Bayesian standardization [14]. Crude rates were log transformed to normality before any subsequent analysis.

#### Environmental risk factors

The occurrence of human leptospirosis infections was compared to biotic and abiotic risk factors derived from a variety of sources and compiled in a single geographic database. Municipality coverage of water infrastructure and land use were obtained from the Dutch national topographic map [15]. Four land use variables were included in the analysis: percentage coverage of arable land, built-up area, grasslands, and forest. Two variables describing surface water infrastructure were included: (1) the total length of narrow waterways in meters per km^2^. Ditches and waterways with < 6 meters were considered narrow waterways. (2) the total length of water banks in meters per km^2^. The total length of water banks was calculated by summing the perimeter of ponds, puddles, lakes, fens and the length of waterways with > 6 meters.

Ground soil coverages were obtained from the Wageningen University DANS [16], which determine soil fertility and ability to retain water, were calculated based on the total surface area (km2) of mineral content of soils within a municipality. Mineral soils were divided into sabulous clay, clay, sand and peat. Clay and sabulous clay were distinguished according to the percentage of lutem (clay) particles with a size of <2μm: heavy sabulous clay (17.5–25%), and heavy clay (>35%).

Municipality population and farm densities were obtained from the Dutch Central Bureau for Statistics [17]. The following variables were included in the subsequent analysis: Population per km^2^ and the number of cattle and cattle/crop combined farms per km^2^. As our data showed that leptospirosis in the Netherlands is most commonly found in people between the age of 45–65 (RR[95%CI] = 1.63[1.31–2.05]), the proportion of population in this age class per municipality was included in the analysis.

### Statistical analysis

#### Disease mapping

Individual case reports showed that of all cases for which a likely location of infection was reported (52%), 83% had the infection contracted within the vicinity of the location of residence. Hotspots of leptospirosis were identified using kernel density estimator using a 15 km bandwidth which was subjectively chosen, informed by the likely place of infection relative to the home address. The log transformed leptospirosis incidence rates were mapped and the existence of spatial autocorrelations between all neighbor pairs was assessed over incremental orders of adjacency. First order neighbor pairs are defined as municipalities directly sharing a border. Likewise, second order neighbors are defined as those separated by one directly adjacent neighbor and so forth. Spatial correlations were calculated using Moran’s I statistic and plotted by the order of adjacency in a spatial correlogram [18]. This approach allows assessing the spatial scale at which leptospirosis incidence rates are clustered. The locations of significant clusters were then identified and mapped using Local Indicators of Spatial Association (LISA) [19].

#### Model fitting and selection

The existence of spatial clusters of increased incidence rates is indicative of a lack of independence between individual municipality observations. To obtain unbiased estimates of the effect of various risk factors this spatial structure was taken into account when modelling the relation between leptospirosis incidences and various risk factors. Simultaneous Auto Regression (SAR) models accounting for spatially auto correlated residual errors were fitted to the municipal incidence rates. SAR models explicitly model the effect of spatial associations by estimating local incidence rates adding a term describing the effect of residual variation observed in neighboring municipalities to the model [20]. This model framework addresses spatial autocorrelation resulting from the omission of spatially heterogeneous environmental predictors allowing for an unbiased inference of coefficient estimates included in the model [20].

To assess the effect of the different environmental variables, each variable was fitted to the data using a univariate SAR model. Next, multivariate models were specified using the full factorial of all cross combinations of eight environmental predictors found significant (p<0.05) in the univariate analysis. This resulted in 255 different models which were fitted to the data. All models were compared based on their fit using the Akaike Information Criterion (AIC) [21]. Since, the best rating model could not be distinguished based merely on the relative AIC values (ΔAIC < 2), the results of the most parsimonious model, (e.g. based on the lowest number of variables) is reported. The final model was then used to estimate leptospirosis incidence rates per municipality. To test whether a model had effectively accounted for non-independence of observations (e.g. spatial autocorrelation) the residual variation was tested for the presence of spatial autocorrelation using Moran’s I.

All descriptive and subsequent spatial analyses were performed using R-statistical language [22]. Outputs were mapped using ArcGIS vs 10.2.

### Ethical issues

Data were collected in compliance with the regulations and principles of the Dutch Public Health Service Policy including ethical consideration (document U05-160JvS.Is). All presented data have been de-identified and are therefore not attributable to individual patients.

## Results and discussion

Out of 536 cases which were laboratory confirmed for leptospirosis in the Netherlands between 1995 and 2012, 236 cases contracted the infection outside the Netherlands. Of the remaining 300 autochthonous cases, 91 cases could not be geo-located to their respective location of infection or residential address. Three cases were identified as infections resulting from contact with pets and were also excluded from the subsequent analysis. In total, 206 autochthonous cases of laboratory confirmed leptospirosis originating from 134 different municipalities were included in the final analysis.

### Spatial pattern of leptospirosis in the Netherlands

**Fig 1:**
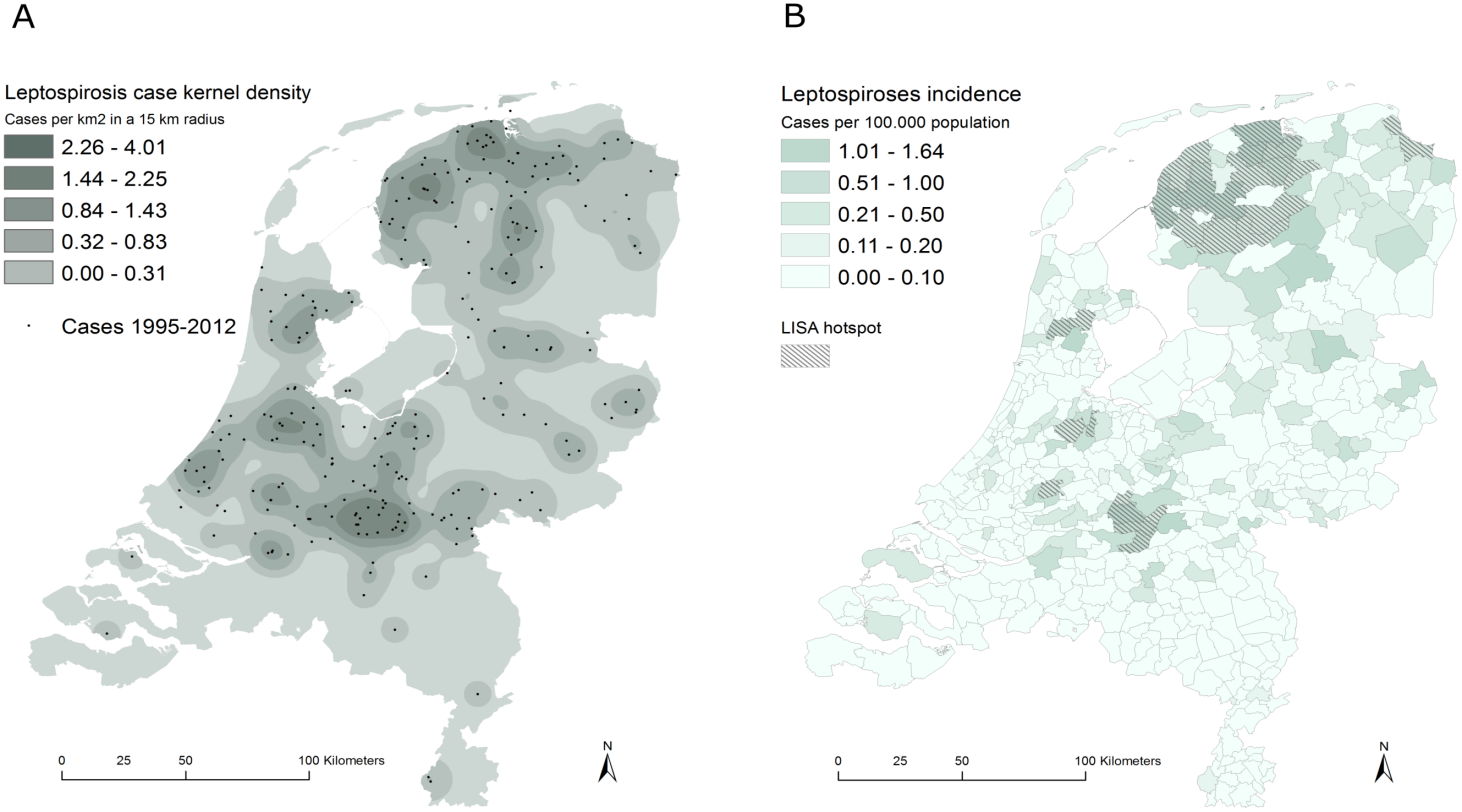
Kernel density estimates. (A) kernel density estimate and point location of Lyme cases and (B) spatial distribution of incidence rates across the Netherlands. Hotpots or clusters of municipalities with increased incidences as identified using the Local Indicators of Spatial Autocorrelation (LISA) analysis are indicated.

**Fig 2:**
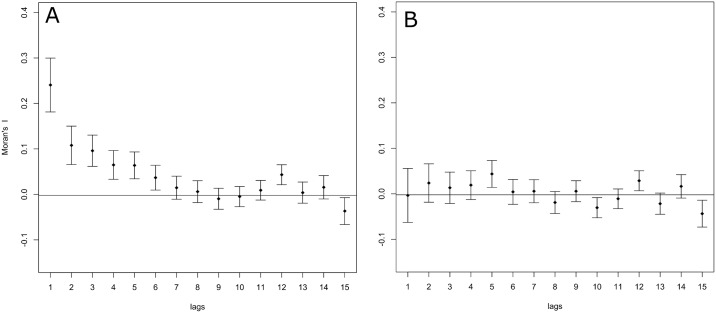
Correlogram showing the spatial auto-correlation of leptospirosis incidence rates. (A) before and (B) after fitting the multivariate model (e.g. residual variation) between municipalities over incremental distances. Lag orders correspond to the order of municipality adjacency ranging from 1^st^ to 15^th^ order neighboring municipalities.

Over the period 1995–2012 the national annual incidence rate was 0.07/100.000 [95%CI: 0.06–0.08]. The pattern of leptospirosis showed a marked geographical pattern and variation between regions (Fig 1a). The kernel density analysis of the case incidences showed clusters of cases to be concentrated in the north and central regions of the Netherlands while low incidences were observed in the south and western part of the country (Fig 1a). The LISA analysis confirmed the presence of seven spatially distinct hotpots of high leptospirosis incidence rates, involving 28 municipalities (Fig 1b). Spatial autocorrelation analysis also showed municipality incidence rates to be correlated up to the 6^th^ order spatial neighbor corresponding to approximately 12.8 km distance [range: 4.8–38.7 km] (Fig 2a).

### Environmental risk factors

Fitting univariate SAR models to the municipal incidence rates showed that leptospirosis incidence rates were significantly associated to eight out of the twelve environmental risk factors fitted to the data. Land-use, soil properties, water-infrastructure, the density of cattle farms and the percentage of population between 45–64 years of age were found to be significantly associated to leptospirosis incidence rates (Table 1). Fitting a spatial SAR model including an intercept only significantly improved model fit over a non-spatial model including an intercept only (LR = 47.29, p = 6.1e-12).

**Table 1:** Overview of the univariate and multivariate effects of environmental predictors to estimate the observed incidence of leptospirosis fitting a spatial auto regression model (SAR) to the data.

Variables	Mean (SD)	Univariate SAR (SE)	Multivariate SAR (SE)
Intercept only	-	-10.65 (0.076)**	-12.1 (0.311)**
Rho (spatial correlation)		0.38 (0.057)**	0.26 (0.062)**
*% Land use*			
Arable land	20.7 (17.3)	0.13 (0.061)^ns^	-0.19 (0.061)*
Built-up area	6.8 (7.0)	-0.58 (0.044)**	-0.55 (0.055)**
Grass	39.3 (18.3)	0.43 (0.053)**	0.16 (0.056)*
Forest	13.1 (11.7)	-0.21 (0.058)**	-
*% Ground soil*			
Heavy sabulous clay	11.0 (14.3)	0.18 (0.06)*	0.17 (0.048)**
Heavy clay	8.0 (13.2)	0.17 (0.06)*	-
Sand	35.2 (36.4)	-0.04 (0.066)^ns^	-
Peat	9.1 (18.3)	0.13 (0.061)^ns^	-
*Water (m/km^2^)*			
Narrow waterways	3714 (2581)	0.26 (0.059)**	-
Water banks	4875 (4348)	-0.03 (0.064)^ns^	-
Farms/km^2^	1.5 (1.0)	0.34 (0.058)**	-
% pop 45–64 yrs of age	26.5(3.55)	0.07(0.008)**	0.05 (0.012)**

Significant:

*(p<0.01),

**(p<0.001)

Multivariate model selection resulted in a final set of seven models with indiscriminate AIC scores (e.g. difference of AIC<2). The final most parsimonious multivariate model included five of the eight variables found to be associated to leptospirosis incidence in the univariate analysis (Table 1). The model showed that leptospirosis was significantly associated with a lower coverage of built-up area (β = -0.55, Z = -9.84), a higher coverage of grassland (β = 0.15, Z = 2.82), a high coverage of heavy sabulous clay in the soil (β = 0.17, Z = 3.52) and the proportion of population between 45–64 years of age (β = 0.15, Z = 2.82). Leptospirosis incidence was found to be negatively correlated to the coverage of arable land (β = -0.19, Z = -3.10). Factors which were found to be significantly associated to leptospirosis in the univariate models but not selected in the final multivariate model were: forest cover, the presence of heavy clay soils, the relative length of narrow waterways and density of cattle farms. Including these factors did not lead to a significant improvement of the model fit suggesting strong correlations with other factors included in the final model (see S1 Fig).

**Fig 3:**
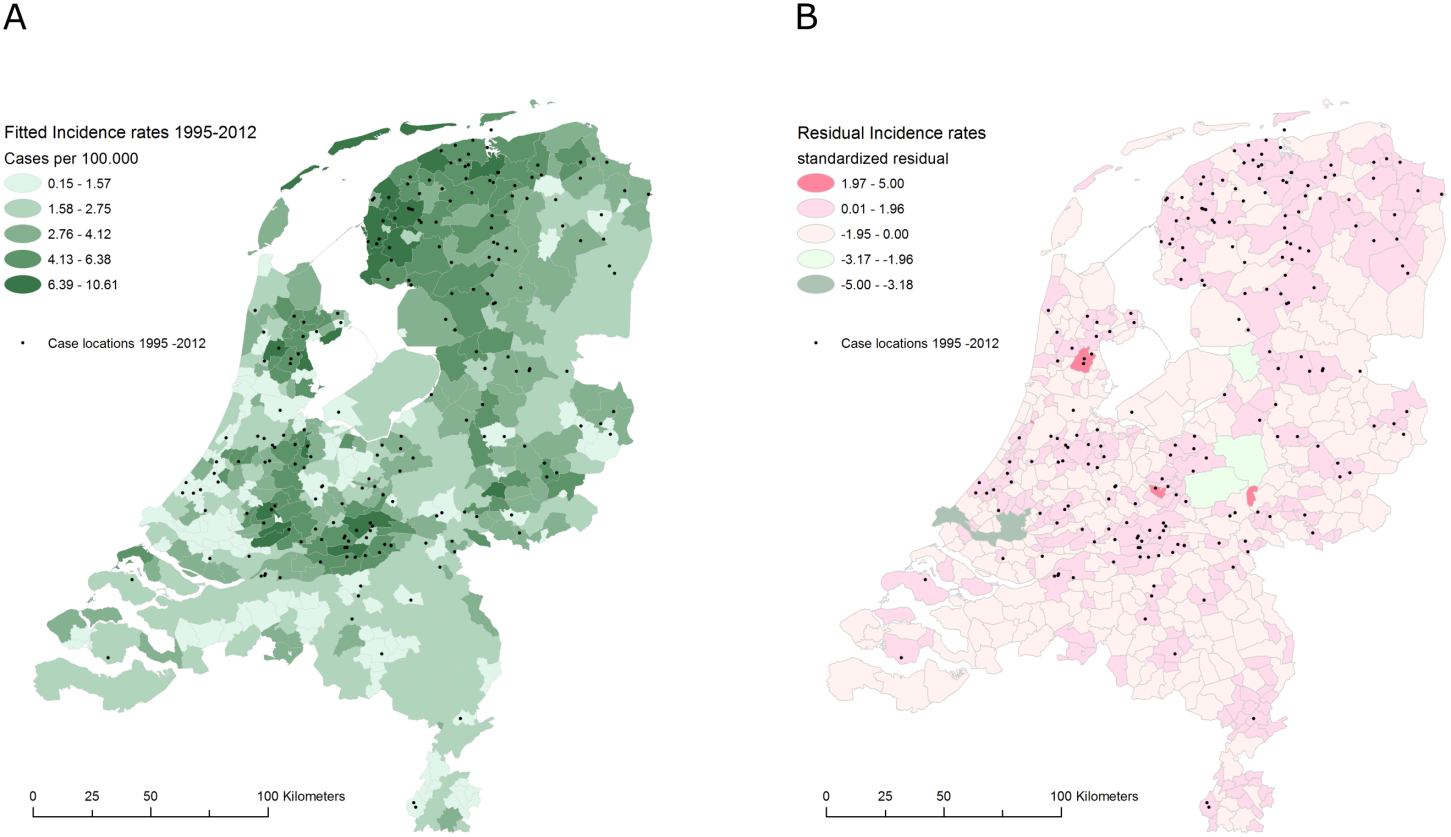
Leptospirosis disease maps of the Netherlands. (A) the estimated leptospirosis incidence and (B) residual variation after fitting the multivariate model to the data.

As observed in the univariate model, accounting for spatial autocorrelation considerably improved the multivariate model fit (ΔAIC = 19.3). Although the effect of the spatial neighborhood decreased compared to a spatial model including an intercept only (ρ = 0.38, z = 6.7), the spatial pattern of leptospirosis was still found to be significantly spatially auto correlated (ρ = 0.28, z = 4.5). This shows that the spatial pattern of leptospirosis is not fully explained by the underlying geographic pattern of risk factors included in the model. Testing residual variation for the presence of spatial autocorrelation showed that no spatial clustering was present in the model residuals after fitting the final model. Hence autocorrelation was effectively accounted for (Figs 2b and 3).

## Discussion

In this study the spatial heterogeneity of leptospirosis was analyzed and associations between the occurrence of the disease and environmental factors were investigated. Both univariate as well as the multivariate analysis showed that leptospirosis was associated with areas depicted by low coverage of built up areas, a high coverage of grassland tenure and high coverage of heavy sabulous clays in the soils. These environmental conditions are typically found in areas of cattle livestock keeping. Surprisingly, the multivariate model did not show this apparent correlation between leptospirosis occurrence and cattle farm density. This is notable considering the fact that farmers present an occupational risk group and outdoor occupation and agricultural activities are commonly associated with increased risk of leptospirosis infection [1,5,6,23]. Over the period 1995–2012, however, farmers in the Netherlands were rarely infected by dairy cattle due to an eradication program for herd infection with serovar Hardjo that was started in 1991 [13]. Therefore, we hypothesize that currently leptospirosis in farmers in the Netherlands is not associated with farm animals as such but is more likely caused by supportive activities outside the farms such as cleaning up ditches and narrow waterways. Moreover, the majority of cattle keeping systems in the Netherlands encompass intensified goat and pig farms which commonly do not have free-ranging animals. Transmission of leptospirosis due to exposure of humans to contaminated natural sources is therefore unlikely. The incidence of human infections is likely to be higher in grassland areas where free ranging cattle keeping systems are common, while this is not indisputably related to cattle farming activities.

Our finding that leptospirosis transmission was higher in areas with high coverage of sabulous clay soils agrees with the results reported by Lau et al. (2012) [24], who conclude that leptospirosis seroprevalence was higher in populations inhabiting areas with loamy clay soils. However, Lau also concludes that leptospirosis seroprevalence is lower on clay soils which contradicts the results of the univariate analysis presented here. In the Netherlands, however, variation in altitude is hardly present, allowing water to inundate on clay soils creating a suitable environment for *Leptospira* to survive [25].

Spatial analysis of the distribution of leptospirosis showed a distinct spatial pattern across the Netherlands. Municipalities with an increased incidence of leptospirosis were found to be clustered up to distances of approximately 12 km. Both univariate and multivariate SAR models showed significant effects of spatial autocorrelation and distinct clusters of leptospirosis which could not solely be explained by the spatial configuration of environmental factors included in these models. It is therefore hypothesized that the observed pattern is a result of both *in situ* environmental conditions facilitating host abundance, exposure and transmission as well increased exposure to contaminated sources within a wider spatial neighborhood resulting in the observed clustering of disease not accounted for by the risk factors in the current model.

Albeit providing valuable insights in the environmental conditions facilitating transmission, several limitations were present in the analyses. Firstly, the number of leptospirosis infections is probably underestimated since mild cases are more likely to go unrecognized. Underreporting of mild clinical case could have resulted in the underestimation of incidence rates in this study. Unequal reporting between different areas could introduce biases in the results. Secondly, cases were geocoded based on their home residence or, if available based on an indication of the assumed location of infection. Imprecisions in geocoding could introduce spatial mismatching and erroneous conclusions about the environment from which infections have emerged. Of all cases for which a likely location of infection was reported (52%), 83% had the infection contracted within 10 km distance of the location of residence. It is therefore expected that spatial mismatches have had a limited impact and were accounted for by the spatial error modeling approach used. The omission of 91 patients (30%) who could not be geolocated is expected to have had a limited impact on these results as no sex, age or temporal bias was observed in these cases. Thirdly we recognize that temporal trends in the emergence of leptospirosis are likely to be present. As the main objective of this study was to investigate spatial patterns in Leptospirosis risk, quantified by using the period incidences, we limited the analysis to investigate spatial trends. Temporal trend in land use could have influenced the results. However shifts in soil are assumed to be unlikely to have occurred over the study period. Similarly no significant shifts in land use were expected to have occurred over the duration of this study [26]

Although, case base point pattern data were initially collected, risk rates were used to be able to compare regional differences in the biotic an abiotic environment to spatial variations in risk. This approach was chosen because: 1) the point location of a household is likely not to reflect the location of infection, 2) aggregation allowed the calculation risk rates using municipality level population data. This approach is a well-established and has been successfully used in the fields of ecology and epidemiology likewise. Although a point pattern analysis could have been used, the use of aggregated data in a SAR modeling framework is believed to produce robust and accurate results.

Since the availability of environmental data is not exhaustive, it is likely that additional environmental factors influencing the occurrence of leptospirosis infection were not examined. Since leptospirosis is mostly transmitted through rodents and especially rats in the Netherlands [13], rodent density is expected to explain the observed spatial distribution of leptospirosis to more detail. Including rat distribution patterns or the spatial configuration of key habitats into the analysis could provide an explanation for the association found between the environmental conditions supporting leptospirosis transmission as they are believed to provide also a suitable habitat for rats and other potential reservoirs. However, reliable data on rodent density in the Netherlands is lacking and high spatial and temporal variation in rodent activity would add considerable complexity the to the model and would require more extensive data on spatial and temporal variations in leptospirosis infections in humans, animals and its prevalence in aquatic and mammal reservoirs.

Estimation of the zoonotic transmission of leptospirosis could favor the use of ecological niche models following Huchinsons fundamental niche concept [27]. To generate a species distribution model [28] for Leptospira spp would require modelling the biotic and abiotic interactions between leptospira and a variety of reservoir host. As outlined in this paper the outcome of interest is the emergence of leptospirosis disease rather than leptospire distribution and abundance. Expectedly, the biotic and abiotic factors presented in this study will determine the distribution of reservoirs host, leptospira survival and ultimately human exposure to the bacterium. The nature and strength of these association, however, will depend on human behavioral factors affecting exposure and ultimately infection. The model presented here is not meant to describe an ecological niche of the pathogen distribution. Rather it is used to estimate risk patterns which are key to effective control and prevention to populations at high risk of disease.

Notwithstanding these limitations to the model presented, the environmental drivers of leptospirosis identified in this study provide valuable information to identify areas of potential transmission and could be used to raise clinical awareness on potential risk of leptospirosis. Deviations of retrospectively observed incidence with respect of the predicted incidence should be investigated to identify additional risk factors required to improve the model (Fig 3).

## Conclusion

The results of this study show that the emergence of Leptospirosis infections in the Netherlands is closely linked to the environment and can be effectively modelled using a combination of soil properties, land-use variables and spatial processes. The observed incidence of leptospirosis was strongly spatially auto correlated with contiguous areas of increased disease incidence of approximately 5–30 km in diameter. This marked pattern could partly be explained by the spatial configuration of environmental risk factors, yet complete spatial dependence was not accounted for by covariate factors. These findings suggest that leptospirosis arises from exposure to ecological conditions facilitating transmission, yet other factors influencing exposure or leptospirosis survival in the environment should be further investigated.

## Supporting information

S1 Fig Dendrogram of covariate correlations. A hierarchical cluster dendrogram was created using between-variable distances calculated as the inverse of pairwise variable correlations (1-ρ).
(DOCX)
