# Supplementary material for: Environmental risk of leptospirosis infections in the Netherlands: Spatial modelling of environmental risk factors of leptospirosis in the Netherlands
Source: PLoS One. 2017 Oct 24;12(10):e0186987. doi: 10.1371/journal.pone.0186987 (PMC5655435; doi:10.1371/journal.pone.0186987)
Supplement: S1 Fig — A hierarchical cluster dendrogram was created using between-variable distances calculated as the inverse of pairwise variable correlations (1-ρ). (DOCX) [file pone.0186987.s001.docx]

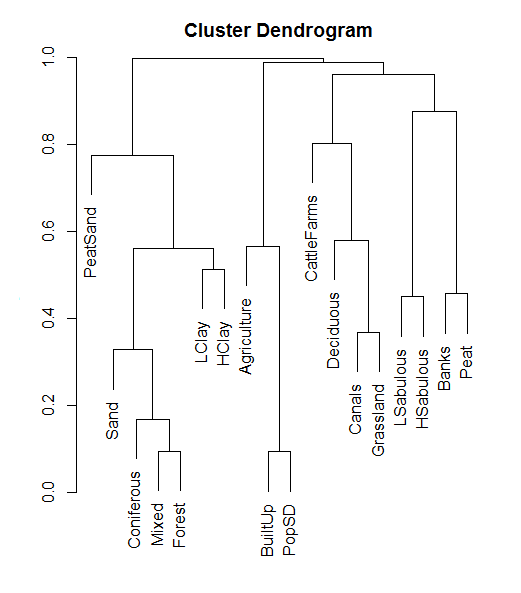
S1 Dendrogram of covariate correlations. A hierarchical cluster dendrogram was created using between-variable distances calculated as the inverse of pairwise variable correlations (1-ρ)
